# The Increasing Financial Impact of Chronic Kidney Disease in Australia

**DOI:** 10.1155/2014/120537

**Published:** 2014-04-01

**Authors:** Patrick S. Tucker, Michael I. Kingsley, R. Hugh Morton, Aaron T. Scanlan, Vincent J. Dalbo

**Affiliations:** ^1^Central Queensland University, Clinical Biochemistry Laboratory, School of Medical and Applied Sciences, Faculty of Sciences, Engineering and Health, Rockhampton, QLD 4702, Australia; ^2^La Trobe University, La Trobe Rural Health School, Faculty of Health Sciences, Bendigo, VIC 3550, Australia; ^3^Massey University, School of Sport and Exercise, Palmerston North 4474, New Zealand

## Abstract

The aim of this investigation was to determine and compare current and projected expenditure associated with chronic kidney disease (CKD), renal replacement therapy (RRT), and cardiovascular disease (CVD) in Australia. Data published by Australia and New Zealand Dialysis and Transplant Registry, Australian Institute of Health and Welfare, and World Bank were used to compare CKD-, RRT-, and CVD-related expenditure and prevalence rates. Prevalence and expenditure predictions were made using a linear regression model. Direct statistical comparisons of rates of annual increase utilised indicator variables in combined regressions. Statistical significance was set at *P* < 0.05. Dollar amounts were adjusted for inflation prior to analysis. Between 2012 and 2020, prevalence, per-patient expenditure, and total disease expenditure associated with CKD and RRT are estimated to increase significantly more rapidly than CVD. RRT prevalence is estimated to increase by 29%, compared to 7% in CVD. Average annual RRT per-patient expenditure is estimated to increase by 16%, compared to 8% in CVD. Total CKD- and RRT-related expenditure had been estimated to increase by 37%, compared to 14% in CVD. Per-patient, CKD produces a considerably greater financial impact on Australia's healthcare system, compared to CVD. Research focusing on novel preventative/therapeutic interventions is warranted.

## 1. Introduction

In 2009-10, Australia spent 121 billion dollars on healthcare, representing a 22.8% increase, adjusted for inflation, compared to expenditure from a decade earlier [1]. In relative terms, healthcare expenditure accounted for 9.4% of Australia's gross domestic product (GDP) in 2009-10, compared to 7.9% of GDP in 1999-00 [1]. Healthcare expenditure is increasing faster than Australia's GDP and, thus, represents an increasingly pressing concern. Contributing to the rise in Australia's total healthcare expenditure is the number of patients undergoing renal replacement therapy (RRT) for chronic kidney disease (CKD), which increased by 81% between 2000 and 2010 [2]. Australia is in the top five of Organisation for Economic Co-operation and Development (OECD) countries (*n* = 34) in terms of life expectancy (81.6 years old) and adults who report being in good health (84.9%) [3]. However, as health-related spending continues to rise, it is becoming increasingly difficult to maintain current levels of healthcare. As a result, it is important to identify the per-patient and total expenditure associated with prevalent conditions such as CKD, with the aim of developing targeted, cost-effective therapeutic interventions that prevent the development and progression of these diseases.

The financial expenditure associated with many of Australia's most prevalent and costly diseases can be reduced by adopting cost-effective lifestyle modifications [4, 5]. Australia's decreasing rate of physical activity is associated with increasing rates of obesity [5, 6], a risk factor that is strongly related to the development and progression of CKD [5, 7, 8]. One large- scale study (*n* = 11, 247) demonstrated that low physical activity levels were strongly associated with obesity [9]. This is important in terms of the Australian population as reports indicate the following: (1) Australia has one of the highest adult obesity rates of the OECD countries (5th of 34), with approximately 25% and 60% of adults classified as obese [6] or overweight [9], respectively, and (2) adult obesity rates have nearly tripled in Australia over the past 20 years, faster than any other OECD country [10]. Assuming that levels of physical activity (currently decreasing) and rates of obesity (currently increasing) continue to follow their current trend in Australia, the prevalence rates of CKD, along with the subsequent CKD-related expenditure, will continue to increase.

Current estimates suggest that approximately 16% of Australian adults have at least one indicator of kidney damage [11] and 10% of Australian deaths have CKD listed as a contributing factor [1]. The 2011 Australia and New Zealand Dialysis and Transplant Registry (ANZDATA) Report indicated that nearly 19,000 Australians were receiving renal replacement therapy (RRT) (life-supporting treatments including hemodialysis, peritoneal dialysis, hemofiltration, and renal transplantation as well as postoperative care) as of December 31, 2010 [12]. Of those patients, 8,409 received a kidney transplant while another 10,590 were currently undergoing dialysis [12]. Due to the substantial expenditure associated with hemodialysis and kidney transplants, which accounts for nearly 85% of total CKD-related expenditure [13], CKD is costly on a per-patient level. Consequently, increasing prevalence rates of CKD are likely to result in a substantial increase to Australia's healthcare expenditure.

Currently, CVD represents the most expensive disease group in Australia [1] and thus serves as a practical benchmark by which to compare the expenditure of other chronic diseases, particularly chronic diseases that share many of the same risk factors such as CKD [14]. As a result, this investigation will determine and compare the financial expenditure produced by CKD, RRT, and CVD with regard to trending and estimation of the following: (1) prevalence rates associated with RRT and CVD; (2) total expenditure associated with CKD, RRT, and CVD and how they compare to total healthcare expenditure; and (3) per-patient expenditure associated with RRT and CVD.

We aim to determine and predict the financial expenditure associated with CKD and RRT, in the hopes of encouraging research designed to develop therapies that reduce the development and progression of CKD, with special consideration given to treatment options that are cost-effective, in order to help keep CKD-related and total Australian healthcare expenditure manageable.

## 2. Materials and Methods 

Data made available by the Australia and New Zealand Dialysis and Transplant Registry (ANZDATA), the Australian Institute of Health and Welfare (AIHW), and World Bank were used to determine and predict the following: (1) Australia's gross domestic product (GDP); (2) total population in Australia; (3) RRT and CVD prevalence in Australia; (4) CKD-, RRT-, and CVD-related healthcare expenditure in Australia; and (5) total healthcare expenditure in Australia. RRT prevalence refers to the number of patients currently undergoing treatments such as hemodialysis, peritoneal dialysis, hemofiltration, and renal transplantation. RRT-related expenditure, the primary contributor to total CKD expenditure, refers to the amount of money spent on the aforementioned life-supporting therapies used to treat renal failure. CKD-related expenditure captures expenditure related to CKD that is beyond RRT related-expenditure (e.g., physician visits and dietary counselling that may or may not take place prior to the initiation of RRT).

### 2.1. Determining Prevalence of RRT and CVD

Total CKD prevalence is not calculated due to the number of undiagnosed patients, as many are asymptomatic and/or undiagnosed during the early stages of CKD. Data published by ANZDATA was used to determine and predict the number of patients undergoing RRT. ANZDATA collects information from all renal-care units in Australia. Data collection occurs at two time points: key events (described as new patients, deaths, and transplants) are reported monthly while an extensive cross-sectional survey is performed yearly. Information published by ANZDATA is based on a final database that is compiled after survey results have been received. Included in the registry are all patients residing in Australia who are receiving long-term RRT. Cases of acute renal failure are excluded.

Data published by AIWH was used to determine and predict CVD prevalence. AIHW reports CVD prevalence rates that are collected and published by the Australian Bureau of Statistics (ABS). Information is obtained by trained ABS interviewers, through a personal computer assisted interview (CAI) using adult members of selected households that fit the scope of the survey. Coding, as it relates to disease state, is undertaken by staff specifically trained for this task. Records are run through an automatic coder. Cases which cannot be coded by autocoders are coded manually using the computer assisted coding (CAC) system. Quality control processes are applied to ensure that the coding process meets acceptable standards.

### 2.2. Determining CKD, RRT, CVD, and Total Healthcare Expenditure

Data published by AIWH was used to determine and predict expenditure associated with CKD, RRT, CVD, and total healthcare. AIHW estimates disease expenditure using a “top-down” approach, where total expenditure across the Australian healthcare system is estimated and then allocated to various diseases. Data on healthcare expenditure are extracted from the AIHW disease expenditure database. Expenditure estimates are then developed using data retrieved from the hospital establishments collection, hospital morbidity records, Medicare, the Pharmaceutical Benefits Scheme (PBS), the Pharmacy Guild Survey, and the BEACH (Bettering the Evaluation and Care of Health) survey of general practice. All dollar amounts are reported in Australia dollars (AUD) and were adjusted for inflation.

### 2.3. Determining Australia's GDP and Total Population

Data published by World Bank was used to determine and predict Australia's GDP and total population. World Development Indicators (WDI) is the primary World Bank database for development data from officially recognized international sources. Global Development Finance (GDF) provides external debt trending and financial flow statistics for countries that report public and publicly guaranteed debt under World Bank's Debtor Reporting System (DRS).

### 2.4. Statistical Analysis

Population, GDP, disease prevalence, and expenditure predictions were made using separate linear regression models and are reported with associated 95% confidence intervals (CI) using SPSS for Windows (V.20.0.0.1, SPSS Inc., Chicago, IL, USA) software. Direct statistical comparisons of the respective rates of annual increase, on a percentage basis, were made using indicator variables in correspondingly combined regressions using Minitab (V.16, Minitab, Inc., Coventry, UK) software. Statistical significance for all tests was set at *P* < 0.05. Dollar amounts were adjusted for inflation prior to analysis. All data were retrieved on June 8, 2013.

## 3. Results

Australian GDP is estimated to increase by 41% between 2012 and 2020 (2012: $1.3 T, 95% CI: $−596.7B–$3.2 T; 2020: $1.8 T, 95% CI: $−1.3 T–$5.0 T) (Table 1). The Australian population is estimated to increase by 10% between 2012 and 2020 (2012: 22.6 M, 95% CI: 18.9 M–26.3 M; 2020: 25.0 M, 95% CI: 18.8 M–31.1 M) (Table 1).

**Figure 1:**
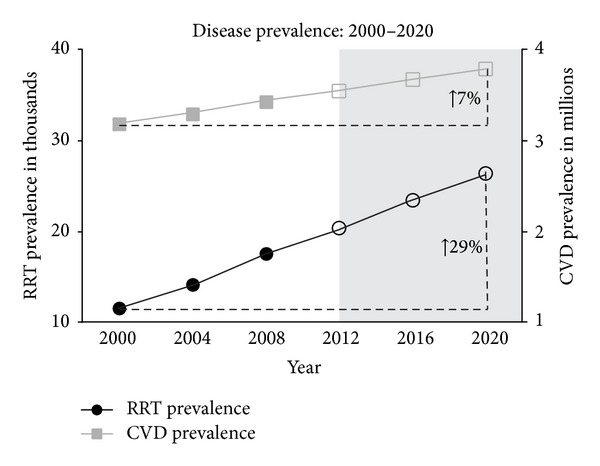


**Figure 2:**
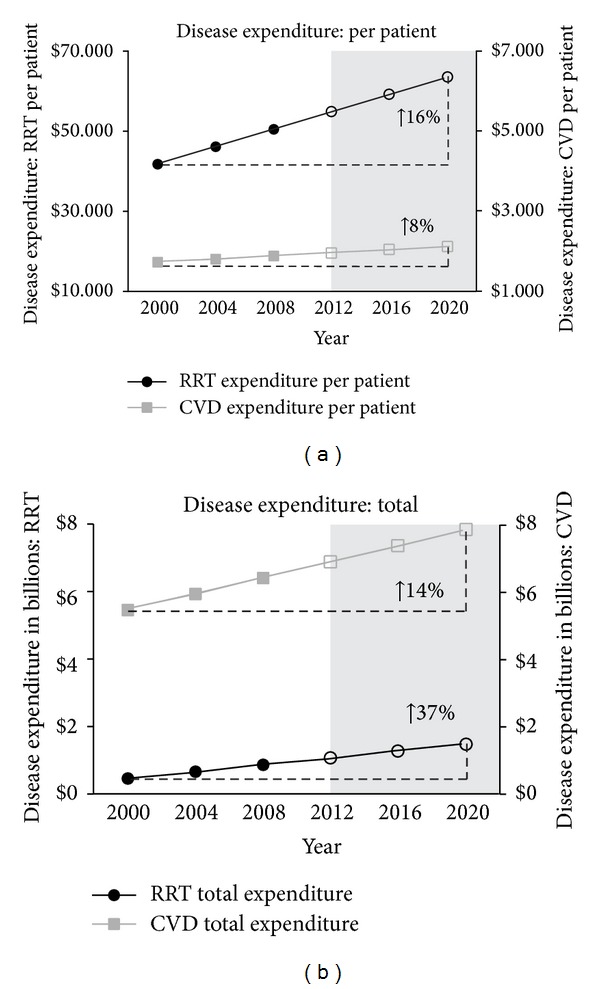


Between 2012 and 2020, RRT prevalence, RRT per-patient expenditure, total CKD expenditure, and total RRT expenditure are estimated to increase significantly more quickly than CVD. RRT prevalence is estimated to increase by 29% (2012: 20,446, 95% CI: 13,504–27,388; 2020: 26,427, 95% CI: 14,811–38,043) compared to a 7% increase in CVD prevalence (2012: 3.5 M, 95% CI: 3.3 M–3.8 M; 2020: 3.8 M, 95% CI: 3.4 M–4.2 M) (*P* < 0.001) (Figure 1). Average annual RRT per-patient expenditure is estimated to increase by 16% (2012: $54,930, 95% CI: $41,738–$68,123; 2020: $63,677, 95% CI: $41,602–$85,753) compared to an 8% increase in average annual CVD per-patient expenditure (2012: $1,954, 95% CI: $1,850–$2,059; 2020: $2,109, 95% CI: $1,935–$2,284) (*P* = 0.002) (Figure 2(a)). CKD- and RRT-related expenditure are estimated to increase by 37% (CKD: 2012: $1.3 B, 95% CI: $381 M–$2.2 B; 2020: $1.7 B, 95% CI: $258 M–$3.2 B) (RRT: 2012: $1.1 B, 95% CI: $324 M–1.8 B; 2020: $1.5 B, 95% CI: $219 M–$2.8 B) compared to a 14% increase in CVD-related expenditure (2012: $6.9 B, 95% CI: $6.6 B–$7.1 B; 2020: $7.8 B, 95% CI: $7.4 B–$8.3 B) (*P* < 0.001) (Figure 2(b)).

**Figure 3:**
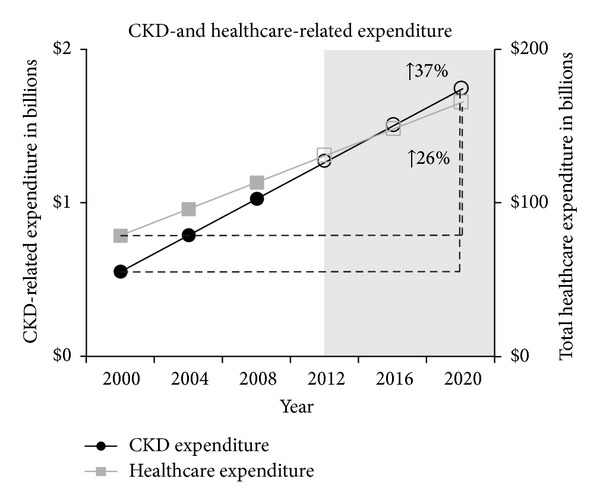


CKD- and RRT-related expenditure are estimated to increase by 37% and 37%, respectively (CKD: 2012: $1.3 B, 95% CI: $381 M–$2.2 B; 2020: $1.7 B, 95% CI: $258 M–$3.2 B) (RRT: 2012: $1.1 B, 95% CI: $324 M–1.8 B; 2020: $1.5 B, 95% CI: $219 M–$2.8 B), compared to a 26% increase in Australia's total healthcare expenditure (2012: $131.4 B, 95% CI: $61.8 B–$201 B; 2020: $166.2 B, 95% CI: $49.7 B–$282.6 B) ( *P* = 0.012) (Figure 3).

## 4. Discussion

This investigation examined (1) the current and predicted prevalence rates of RRT and CVD and (2) the current and predicted expenditure associated with CKD-, RRT-, and CVD-related healthcare in Australia. Current and predicted expenditure had been then used to make comparisons between diseases as well as associated factors such as total healthcare expenditure, GDP, and population growth in Australia. Healthcare expenditure associated with RRT is increasing at a faster rate than the healthcare expenditure associated with CVD due, in part, to the fact that RRT prevalence is increasing at a faster rate than CVD prevalence. CKD and CVD share many of the same risk factors and can be considered as risk factors for one another [15]. Aside from sharing risk factors such as obesity, high blood pressure, poor glycemic control, and low levels of physical activity, CKD increases the risk of complications due to CVD. Thus, it stands to reason that as incidence/prevalence rates of CKD increase, so will the incidence/prevalence rates of CVD. However, this does not explain the disproportionate increase in prevalence rates of RRT, compared to prevalence rates of CVD.

One explanation for the disproportionate increase in prevalence rates of RRT may be that patients with CVD have begun surviving longer due to advances in cardiovascular medicine, giving rise to the development of subsequently diagnosed comorbidities such as CKD. For instance, in 2009, approximately 63% of patients who suffered myocardial infarction survived, compared to 47% in 1997 [6]. Moreover, age standardized CVD death rates have been on the decline since the late 1960's in Australia [6]. This is noteworthy when considered in addition to the fact that CVD prevalence is increasing. Extending the life of patients with CVD increases the likelihood that patients will develop diagnosed kidney disease. It is important to distinguish between diagnosed and undiagnosed CKD as many patients with CVD have some degree of undiagnosed CKD which may exacerbate CVD-related risk factors such as high blood pressure.

Another proposal that may explain the disproportionate increase in RRT prevalence, compared to CVD prevalence, is that healthcare practitioners are becoming more vigilant in regard to diagnosing CKD, given the increased understanding of the relationship between CKD and other prevalent diseases such as CVD [16] and type II diabetes [17]. Research conducted over the past decade has established the exacerbatory relationship between CKD and CVD which has led to the earlier diagnosis and subsequent treatment of CKD in patients that present with CVD-related risk factors. The earlier diagnosis and subsequent treatment of CKD may be partially responsible for the inordinate rise in RRT prevalence and RRT-related expenditure, relative to CVD. The earlier detection and treatment of CKD may, in turn, be partially responsible for the decline in CVD-related deaths as improved control of kidney function would serve to help ameliorate some complications associated with CVD such as hypertension [18] and oxidative stress [19, 20]. Nonetheless, this does not diminish the fact that CKD-related expenditure is increasing at a disproportionate rate, relative to CVD. It can be speculated that if every currently existing case of CKD that warranted treatment were (simultaneously) diagnosed, the resultant spike in CKD-related expenditure would be substantial as it is estimated that over three million Australians currently have at least one indicator of kidney damage [11, 21].

In addition to increasing incidence rates of RRT, increasing prevalence rates also contribute to the growing financial expenditure associated with CKD. The number of patients treated with dialysis or living with a kidney transplant in Australia has increased dramatically over the past three decades (from 2,200 in 1977 to 18,300 in 2009, a 731% increase) [6], and the number continues to grow as a result of increasing incidence and improved survival rates. Dialysis [22] and kidney transplant [23] procedures have become more sophisticated over the past decade and, as a result, patients with late-stage CKD survive longer than they did in the past. As survival rates increase, so will the expenditure associated with ongoing treatment such as dialysis and postoperative therapy following renal transplantation.

It is worthwhile to mention that hypertension is the most frequently managed condition by Australian general practitioners, accounting for 8.7% of patient interactions in 2010-11 [6]. In fact, hypertension is responsible for more deaths than any other biomedical risk factor, worldwide [24]. This partially explains CVD's high prevalence rate, as hypertension is Australia's largest contributor to CVD. It also partially explains CVD's substantial contribution to total healthcare expenditure as hypertension (and its treatment) is classified as a CVD-related expenditure (per the International Statistical Classification of Diseases and Related Health Problems, a medical classification list produced by the World Health Organization) [25]. The classification of hypertension as “CVD-related” is one of the primary reasons why the total CVD-related expenditure is far greater than total CKD-related expenditure. However, hypertension plays an important role in CKD development and progression, is one of the primary risk factors for CKD, and is closely related to kidney function [18]. Thus, the mutual relationship between hypertension and CKD should not be overlooked. A large investigation (*n* = 8,927) in predialysis CKD patients (patients that have yet to undergo dialysis procedures or kidney transplants) found that 67% were hypertensive [18]. Furthermore, this study found that as CKD progressed, blood pressure increased [18]. This supports the notion that patients may be treated for hypertension (commonly categorized as “CVD-related,” for hospital coding purposes) when, concurrently, they are suffering from some undisclosed level of kidney dysfunction. As such, it stands to reason that estimates of CKD prevalence and CKD-related expenditure may be grossly underestimated. Due to shortcomings in current coding procedures, the true prevalence and cost of CKD are not apparent in the absence of traditional CKD-related therapies such as hemodialysis, renal transplantation, or postrenal-transplant therapies.

In the present study, the large discrepancy between total CKD-related and CVD-related healthcare expenditure is a consequence of disease prevalence. According to current estimates, over 3.5 million patients have CVD while just over 20,000 patients are undergoing RRT. Nevertheless, results from this study clearly demonstrate that CKD produces a greater financial impact than CVD, when examined on a per-patient basis. The average RRT-related per-patient expenditure is roughly 27 times greater than the average CVD-related per-patient expenditure ($50,557 per RRT patient versus $1,877 per CVD patient in 2008). Moreover, RRT per-patient expenditure is increasing at a faster rate than CVD per-patient expenditure, indicating that this discrepancy will become larger over time. Projected per-patient expenditure will reach $63,677 per RRT patient versus $2,109 per CVD patient by 2020, representing average RRT-related per-patient expenditure that is over 30 times greater than average CVD-related per-patient expenditure. Notably, the per-patient RRT-related expenditure and projected RRT-related expenditure discussed in the present study are in agreement with data published in a 2010 report by Kidney Health Australia [2].

In terms of cost-effective therapies for patients with CKD or CKD-related risk factors, lifestyle interventions such as weight loss, structured diet, and exercise have emerged as promising and practical options. Weight gain is significantly related to CKD development [26], even in the absence of other contributing factors such as metabolic syndrome [8]. Structured dietary strategies reduce the incidence of CKD [27], reduce metabolic acidosis [28], reduce kidney injury [28], and improve eGFR [29]. Exercise reduces oxidative stress [30], reduces serum alkaline phosphatase [30], improves HDL-C levels [31], improves eGFR [31], and improves cardiovascular reactivity [32]. One study combined the three aforementioned strategies (weight management, structured diet, and exercise) and found that the management of body weight, facilitated by a structured diet and an exercise intervention, resulted in significant improvements to blood pressure control, lipid profiles, serum creatinine levels, eGFR, and proteinuria [5]. The interested reader is urged to consult several compelling reviews that examine the effects of dietary interventions [33, 34], weight management [33, 35], and exercise [36, 37] on CKD.

Inherent uncertainties exist when making projections. Increases in the prevalence of RRT may not continue in such a steady fashion as it has been since 2000. However, there is no existing evidence to suggest this is the case. If current trends continue undeterred, an increase in the prevalence of RRT (and the subsequent increase in financial expenditure associated with it) can be confidently assumed. The availability of appropriate data is also a limitation of the current study. Trending in this study was based on three previous data points: 2000, 2004, and 2008. This was unavoidable for two reasons: (1) often times, data of this nature is compiled less often than annually; (2) hospital coding procedures have changed over time, making direct comparisons to data collected prior to 2000 untenable in the current study. Indeed, data coding procedures may change again in the future and researchers are encouraged to carefully examine the procedures used to collect and code data, prior to initiating this type of study. Furthermore, predictions concerning CKD prevalence and CKD-related financial expenditure must be cautiously analysed as the true prevalence and cost of CKD had been masked by the fact that failing health due to CKD may not be coded/categorized as CKD-related if the disease has not been formally diagnosed. Considering this, it can be assumed that the true prevalence and cost of CKD are greater than those suggested by current data.

## 5. Conclusions

The substantial healthcare expenditure associated with CVD is due to the large number of patients who suffer from this category of disease. CKD often goes undiagnosed in the early stages of the disease, causing cost-relation estimates to underestimate the economic impact of CKD. When examined on a per-patient basis, CKD produces a considerably greater impact on Australia's healthcare system, compared to CVD. Given the high per-patient expenditure and increasing prevalence of CKD, research focusing on novel prevention and/or therapeutic interventions is warranted. Lifestyle interventions, such as weight management, structured diet, and exercise training, may prove to be cost-effective means of therapy for CKD patients. Research that focuses on the direct effects of these interventions on kidney function and kidney function's relationship to cardiovascular risk factors is encouraged.

## Figures and Tables

**Table 1:** Current and projected prevalence rates and expenditure in Australia: 2012–2020.

	2012	2020	Percent Increase
RRT Prevalence	20,446	26,427	29%
CVD Prevalence	3.5 M	3.8 M	7%
CKD-Related Expenditure	$1.3 B	$1.7 B	37%
RRT-Related Expenditure	$1.1 B	$1.5 B	37%
CVD-Related Expenditure	$6.9 B	$7.8 B	14%
Total Healthcare Expenditure	$131 B	$166 B	26%
RRT Per-Patient Expenditure	$54,930	$63,677	16%
CVD Per-Patient Expenditure	$1,954	$2,109	8%
Gross Domestic Product	$1.3 T	$1.8 T	41%
AU Population	$22.6 M	$25.0 M	10%
